# 112 Computer Adaptive Test Development of Preschool Life Impact Burn Recovery Evaluation

**DOI:** 10.1093/jbcr/irad045.085

**Published:** 2023-05-15

**Authors:** Khushbu Patel, Pengsheng Ni, Kate Surette, Madeleine McGwin, Petra Warner, Renata Fabia, Carrie Tully, Jeffrey Schneider, Lewis Kazis, Colleen Ryan, Frederick Stoddard, Tina Palmieri

**Affiliations:** Shriners Children's Boston, Massachusetts General Hospital, Boston, Massachusetts; Boston University School of Public Health, Boston, Massachusetts; Shriners Children's - Boston, Boston, Massachusetts; Shriners Children's- Boston, Boston, MA, Boston, Massachusetts; Shriners Children's Ohio, dayton, Ohio; Nationwide Children's Hospital, Columbus, Ohio; Children's National Hospital, Washington, District of Columbia; Spaulding Rehabilitation Hospital/Harvard Medical School/Rehabilitation Outcomes Center at Spaulding, Boston, Massachusetts; Boston University School of Public Health, Rehabilitation Outcomes Center at Spaulding, Boston, Massachusetts; Mass Gen Hosp/Harvard, Shriners Children's Boston, Boston, Massachusetts; Harvard Medical School at the Massachusetts General Hospital, Boston, Massachusetts; UC Davis and Shriners Children's Northern California, Sacramento, California; Shriners Children's Boston, Massachusetts General Hospital, Boston, Massachusetts; Boston University School of Public Health, Boston, Massachusetts; Shriners Children's - Boston, Boston, Massachusetts; Shriners Children's- Boston, Boston, MA, Boston, Massachusetts; Shriners Children's Ohio, dayton, Ohio; Nationwide Children's Hospital, Columbus, Ohio; Children's National Hospital, Washington, District of Columbia; Spaulding Rehabilitation Hospital/Harvard Medical School/Rehabilitation Outcomes Center at Spaulding, Boston, Massachusetts; Boston University School of Public Health, Rehabilitation Outcomes Center at Spaulding, Boston, Massachusetts; Mass Gen Hosp/Harvard, Shriners Children's Boston, Boston, Massachusetts; Harvard Medical School at the Massachusetts General Hospital, Boston, Massachusetts; UC Davis and Shriners Children's Northern California, Sacramento, California; Shriners Children's Boston, Massachusetts General Hospital, Boston, Massachusetts; Boston University School of Public Health, Boston, Massachusetts; Shriners Children's - Boston, Boston, Massachusetts; Shriners Children's- Boston, Boston, MA, Boston, Massachusetts; Shriners Children's Ohio, dayton, Ohio; Nationwide Children's Hospital, Columbus, Ohio; Children's National Hospital, Washington, District of Columbia; Spaulding Rehabilitation Hospital/Harvard Medical School/Rehabilitation Outcomes Center at Spaulding, Boston, Massachusetts; Boston University School of Public Health, Rehabilitation Outcomes Center at Spaulding, Boston, Massachusetts; Mass Gen Hosp/Harvard, Shriners Children's Boston, Boston, Massachusetts; Harvard Medical School at the Massachusetts General Hospital, Boston, Massachusetts; UC Davis and Shriners Children's Northern California, Sacramento, California; Shriners Children's Boston, Massachusetts General Hospital, Boston, Massachusetts; Boston University School of Public Health, Boston, Massachusetts; Shriners Children's - Boston, Boston, Massachusetts; Shriners Children's- Boston, Boston, MA, Boston, Massachusetts; Shriners Children's Ohio, dayton, Ohio; Nationwide Children's Hospital, Columbus, Ohio; Children's National Hospital, Washington, District of Columbia; Spaulding Rehabilitation Hospital/Harvard Medical School/Rehabilitation Outcomes Center at Spaulding, Boston, Massachusetts; Boston University School of Public Health, Rehabilitation Outcomes Center at Spaulding, Boston, Massachusetts; Mass Gen Hosp/Harvard, Shriners Children's Boston, Boston, Massachusetts; Harvard Medical School at the Massachusetts General Hospital, Boston, Massachusetts; UC Davis and Shriners Children's Northern California, Sacramento, California; Shriners Children's Boston, Massachusetts General Hospital, Boston, Massachusetts; Boston University School of Public Health, Boston, Massachusetts; Shriners Children's - Boston, Boston, Massachusetts; Shriners Children's- Boston, Boston, MA, Boston, Massachusetts; Shriners Children's Ohio, dayton, Ohio; Nationwide Children's Hospital, Columbus, Ohio; Children's National Hospital, Washington, District of Columbia; Spaulding Rehabilitation Hospital/Harvard Medical School/Rehabilitation Outcomes Center at Spaulding, Boston, Massachusetts; Boston University School of Public Health, Rehabilitation Outcomes Center at Spaulding, Boston, Massachusetts; Mass Gen Hosp/Harvard, Shriners Children's Boston, Boston, Massachusetts; Harvard Medical School at the Massachusetts General Hospital, Boston, Massachusetts; UC Davis and Shriners Children's Northern California, Sacramento, California; Shriners Children's Boston, Massachusetts General Hospital, Boston, Massachusetts; Boston University School of Public Health, Boston, Massachusetts; Shriners Children's - Boston, Boston, Massachusetts; Shriners Children's- Boston, Boston, MA, Boston, Massachusetts; Shriners Children's Ohio, dayton, Ohio; Nationwide Children's Hospital, Columbus, Ohio; Children's National Hospital, Washington, District of Columbia; Spaulding Rehabilitation Hospital/Harvard Medical School/Rehabilitation Outcomes Center at Spaulding, Boston, Massachusetts; Boston University School of Public Health, Rehabilitation Outcomes Center at Spaulding, Boston, Massachusetts; Mass Gen Hosp/Harvard, Shriners Children's Boston, Boston, Massachusetts; Harvard Medical School at the Massachusetts General Hospital, Boston, Massachusetts; UC Davis and Shriners Children's Northern California, Sacramento, California; Shriners Children's Boston, Massachusetts General Hospital, Boston, Massachusetts; Boston University School of Public Health, Boston, Massachusetts; Shriners Children's - Boston, Boston, Massachusetts; Shriners Children's- Boston, Boston, MA, Boston, Massachusetts; Shriners Children's Ohio, dayton, Ohio; Nationwide Children's Hospital, Columbus, Ohio; Children's National Hospital, Washington, District of Columbia; Spaulding Rehabilitation Hospital/Harvard Medical School/Rehabilitation Outcomes Center at Spaulding, Boston, Massachusetts; Boston University School of Public Health, Rehabilitation Outcomes Center at Spaulding, Boston, Massachusetts; Mass Gen Hosp/Harvard, Shriners Children's Boston, Boston, Massachusetts; Harvard Medical School at the Massachusetts General Hospital, Boston, Massachusetts; UC Davis and Shriners Children's Northern California, Sacramento, California; Shriners Children's Boston, Massachusetts General Hospital, Boston, Massachusetts; Boston University School of Public Health, Boston, Massachusetts; Shriners Children's - Boston, Boston, Massachusetts; Shriners Children's- Boston, Boston, MA, Boston, Massachusetts; Shriners Children's Ohio, dayton, Ohio; Nationwide Children's Hospital, Columbus, Ohio; Children's National Hospital, Washington, District of Columbia; Spaulding Rehabilitation Hospital/Harvard Medical School/Rehabilitation Outcomes Center at Spaulding, Boston, Massachusetts; Boston University School of Public Health, Rehabilitation Outcomes Center at Spaulding, Boston, Massachusetts; Mass Gen Hosp/Harvard, Shriners Children's Boston, Boston, Massachusetts; Harvard Medical School at the Massachusetts General Hospital, Boston, Massachusetts; UC Davis and Shriners Children's Northern California, Sacramento, California; Shriners Children's Boston, Massachusetts General Hospital, Boston, Massachusetts; Boston University School of Public Health, Boston, Massachusetts; Shriners Children's - Boston, Boston, Massachusetts; Shriners Children's- Boston, Boston, MA, Boston, Massachusetts; Shriners Children's Ohio, dayton, Ohio; Nationwide Children's Hospital, Columbus, Ohio; Children's National Hospital, Washington, District of Columbia; Spaulding Rehabilitation Hospital/Harvard Medical School/Rehabilitation Outcomes Center at Spaulding, Boston, Massachusetts; Boston University School of Public Health, Rehabilitation Outcomes Center at Spaulding, Boston, Massachusetts; Mass Gen Hosp/Harvard, Shriners Children's Boston, Boston, Massachusetts; Harvard Medical School at the Massachusetts General Hospital, Boston, Massachusetts; UC Davis and Shriners Children's Northern California, Sacramento, California; Shriners Children's Boston, Massachusetts General Hospital, Boston, Massachusetts; Boston University School of Public Health, Boston, Massachusetts; Shriners Children's - Boston, Boston, Massachusetts; Shriners Children's- Boston, Boston, MA, Boston, Massachusetts; Shriners Children's Ohio, dayton, Ohio; Nationwide Children's Hospital, Columbus, Ohio; Children's National Hospital, Washington, District of Columbia; Spaulding Rehabilitation Hospital/Harvard Medical School/Rehabilitation Outcomes Center at Spaulding, Boston, Massachusetts; Boston University School of Public Health, Rehabilitation Outcomes Center at Spaulding, Boston, Massachusetts; Mass Gen Hosp/Harvard, Shriners Children's Boston, Boston, Massachusetts; Harvard Medical School at the Massachusetts General Hospital, Boston, Massachusetts; UC Davis and Shriners Children's Northern California, Sacramento, California; Shriners Children's Boston, Massachusetts General Hospital, Boston, Massachusetts; Boston University School of Public Health, Boston, Massachusetts; Shriners Children's - Boston, Boston, Massachusetts; Shriners Children's- Boston, Boston, MA, Boston, Massachusetts; Shriners Children's Ohio, dayton, Ohio; Nationwide Children's Hospital, Columbus, Ohio; Children's National Hospital, Washington, District of Columbia; Spaulding Rehabilitation Hospital/Harvard Medical School/Rehabilitation Outcomes Center at Spaulding, Boston, Massachusetts; Boston University School of Public Health, Rehabilitation Outcomes Center at Spaulding, Boston, Massachusetts; Mass Gen Hosp/Harvard, Shriners Children's Boston, Boston, Massachusetts; Harvard Medical School at the Massachusetts General Hospital, Boston, Massachusetts; UC Davis and Shriners Children's Northern California, Sacramento, California; Shriners Children's Boston, Massachusetts General Hospital, Boston, Massachusetts; Boston University School of Public Health, Boston, Massachusetts; Shriners Children's - Boston, Boston, Massachusetts; Shriners Children's- Boston, Boston, MA, Boston, Massachusetts; Shriners Children's Ohio, dayton, Ohio; Nationwide Children's Hospital, Columbus, Ohio; Children's National Hospital, Washington, District of Columbia; Spaulding Rehabilitation Hospital/Harvard Medical School/Rehabilitation Outcomes Center at Spaulding, Boston, Massachusetts; Boston University School of Public Health, Rehabilitation Outcomes Center at Spaulding, Boston, Massachusetts; Mass Gen Hosp/Harvard, Shriners Children's Boston, Boston, Massachusetts; Harvard Medical School at the Massachusetts General Hospital, Boston, Massachusetts; UC Davis and Shriners Children's Northern California, Sacramento, California

## Abstract

**Introduction:**

Measuring physical, psychological, and social outcomes following childhood burn injury is important to understand the impact of the burn. Condition-specific outcomes are highly relevant in assessments for purposes of gauging early growth and development through parent-reported outcome measures (PROMs). Item content in existing PROMs for preschool children is limited and often covers only one construct. Computer adaptive tests (CATs) with advanced psychometric artificial intelligence technologies minimizes respondent burden and enables the creation of a comprehensive condition-specific instrument. This project developed a parent-reported CAT (Preschool_1-5_ Life Impact Burn Recovery Evaluation (PS-LIBRE_1-5_) Profile) to measure relevant outcomes after burn injuries in children aged one to under six years.

**Methods:**

Responses to the field-tested PS-LIBRE_1-5_ Profile (188 items) were measured on a scale of frequency or ability levels. Scores were coded from 0 to 4 where higher scores reflected better functioning. Factor analysis and Partial Credit Models identified the items retained in the final item bank of each scale. Differential item functioning (DIF) determined if subgroups of burn survivors at the same ability level demonstrated different probabilities of responses. CAT simulations were conducted to estimate the mean score of each scale. The simulated CAT score and full item bank scores were compared based upon the score range, ceiling and floor effects, and marginal reliabilities.

**Results:**

The child mean age was 3.0 + 1.5 years (n = 498), with primarily mothers (83%) responding. Average burn size and time since burn injury were 4.2% TBSA and 1.1 years, respectively. Analysis resulted in eight scales: Physical (27 items), Communication and language (20 items), Emotional wellbeing (16 items), Mood (11 items), Anxiety (13 items), Peer acceptance (7 items), Play (8 items), and Peer relations (9 items). Severe DIF by ‘age at survey completion’ was shown in 13 items. Ceiling effects were acceptable at < 14%. A CAT was created with the exception of four of the scales (Peer acceptance, Play, Mood, and Anxiety) due to limited item content in these scales’ full item bank. Marginal reliabilities were credible (0.83 to 0.90). CAT simulations revealed that administering the CAT would save the respondent about 14 minutes compared to administering the full item bank.

**Conclusions:**

Final scales of the PS-LIBRE_1-5_ Profile contain 111 items and is a comprehensive measure that captures physical, communication and language, psychological, and social functioning of preschool burn survivors in the community.

**Applicability of Research to Practice:**

This work lays the groundwork for developing a set of age-based CATs, adding to prior adult LIBRE project. The CAT aims to detect the progress of a child during burn recovery for use in clinical practice.

